# Supplementary material for: Single nucleus transcriptomics of ventral midbrain identifies glial activation associated with chronic opioid use disorder
Source: Nat Commun. 2023 Sep 12;14:5610. doi: 10.1038/s41467-023-41455-8 (PMC10497570; doi:10.1038/s41467-023-41455-8)
Supplement: Supplementary file 1 — Supplementary Information [file 41467_2023_41455_MOESM1_ESM.pdf]

SUPPLEMENTARY MATERIALS FOR

Single nucleus transcriptomics of ventral midbrain identifies glial activation associated with chronic opioid use disorder

Julong Wei, Tova Y. Lambert, Aditi Valada, Nikhil Patel, Kellie Walker, Jayna Lenders, Carl J. Schmidt, Marina Iskhakova, Adnan Alazizi, Henriette Mair-Meijers, Deborah C. Mash, Francesca Luca, Roger Pique-Regi, Michael J Bannon, Schahram Akbarian

This supplementary file includes Figure S1- S10 and Table S1

Figure S1: Quality controls for VM single nuclei RNA-seq profiling.

Figure S2: Distribution of single nuclei with astrocyte, oligodendrocyte (ODC) and neuronal gene expression.

Figure S3: Cell type-specific expression of opioid receptor genes in the VM.

Figure S4: Differential gene expression across cohorts and cell types.

Figure S5: Opioid exposure affects the transcriptome of multiple subtypes of oligodendrocytes and microglia.

Figure S6: Cell-type specific pathway enrichments of differentially expressed genes in astrocytes and microglia.

Figure S7: Cell-type specific pathway enrichments of differentially expressed genes in oligodendrocytes (ODC) and non-dopaminergic neurons (non-DA).

Figure S8: Correlations between prior bulk RNA-seq study and the current RNA-seq analysis with single cell resolution.

Figure S9: Differentially expressed genes (DEGs) linked to Transcriptome Wide Association Studies (TWAS).

Figure S10: Tissue and demographic variables impacting single nuclei transcriptomes.

Table S1: Summary of Case and Control Cohort (Demographics and postmortem confounds)

Supplementary References

Figure S1

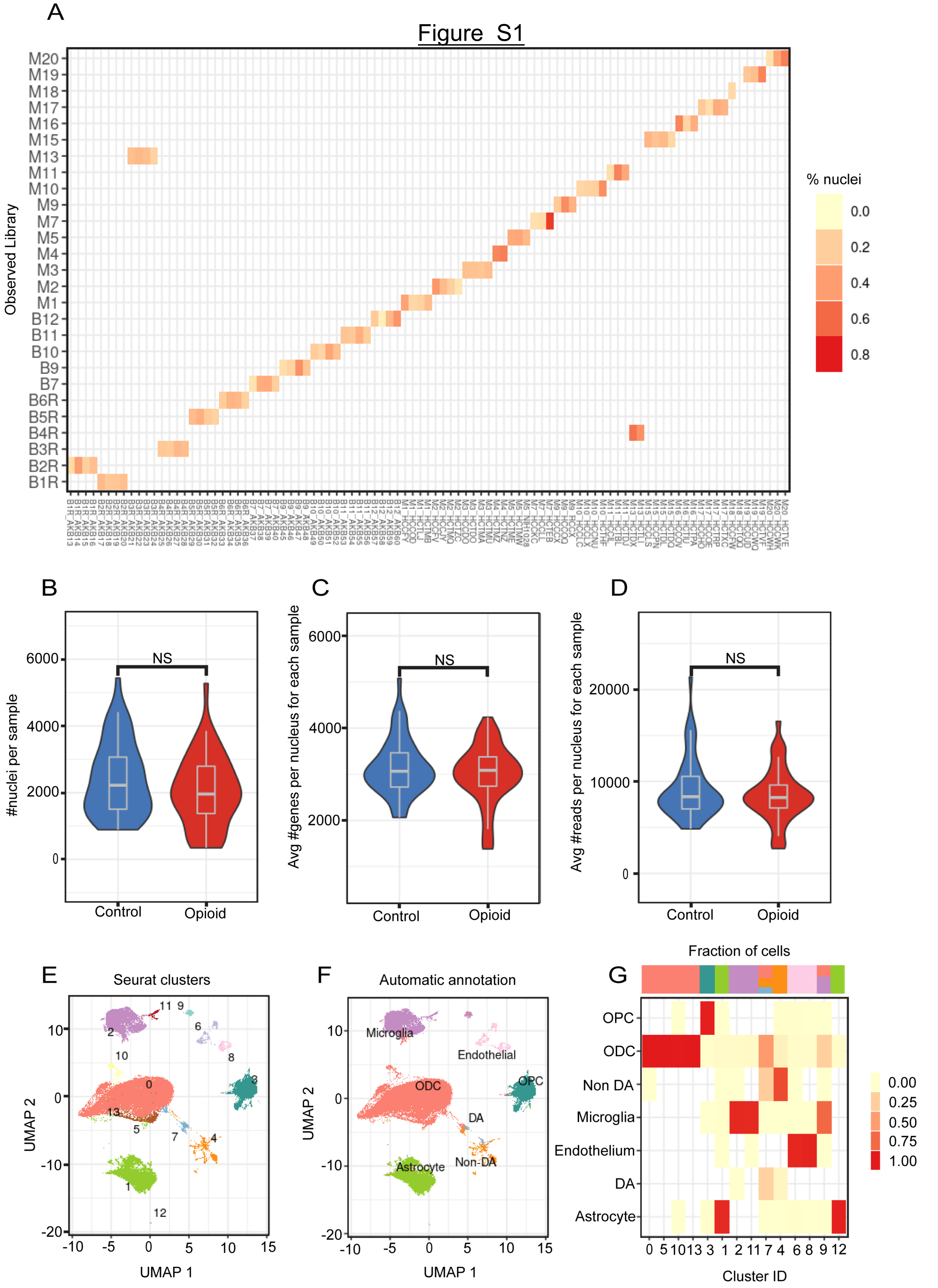
A
Observed Library
M20
M19
M18
M17
M16
M15
M13
M11
M10
M9
M7
M5
M4
M3
M2
M1
B12
B11
B10
B9
B7
B6R
B5R
B4R
B3R
B2R
B1R
% nuclei
0.0
0.2
0.4
0.6
0.8
B
#nuclei per sample
NS
Control
Opioid
C
Avg #genes per nucleus for each sample
NS
Control
Opioid
D
Avg #reads per nucleus for each sample
NS
Control
Opioid
E
Seurat clusters
UMAP 1
UMAP 2
F
Automatic annotation
Microglia
Endothelial
ODC
OPC
DA
Astrocyte
Non-DA
UMAP 1
UMAP 2
G
Fraction of cells
OPC
ODC
Non DA
Microglia
Endothelium
DA
Astrocyte
0.00
0.25
0.50
0.75
1.00
0 5 10 13 3 1 2 11 7 4 6 8 9 12
Cluster ID

Figure S1: Quality controls for VM single nuclei RNA-seq profiling. (A) Dexmulet genotyping confirms 100% match by donor by pool against the background of all 95 donors. Y-axis represents the library pools for 95 individuals. X axis represents the individual, prefix of which denoting the expected library for each subject. Heat bar, percentage of nuclei for specific individual in the library, as indicated. (B-D) Violin plots, showing separately for 45 individuals who died by opioid overdose (blue) and 50 controls (red), (B) the distribution of numbers of nuclei per subject (1 sample = 1 subject), (C) the average number of genes per nucleus for each subject, and (D) the average numbers of reads per sample (1 sample = 1 subject). (E) UMAP plot for VM from 95 samples (present) study, (F, G) automatic annotation of major VM cell types from previous study in a smaller reference sample[1].

Figure S2

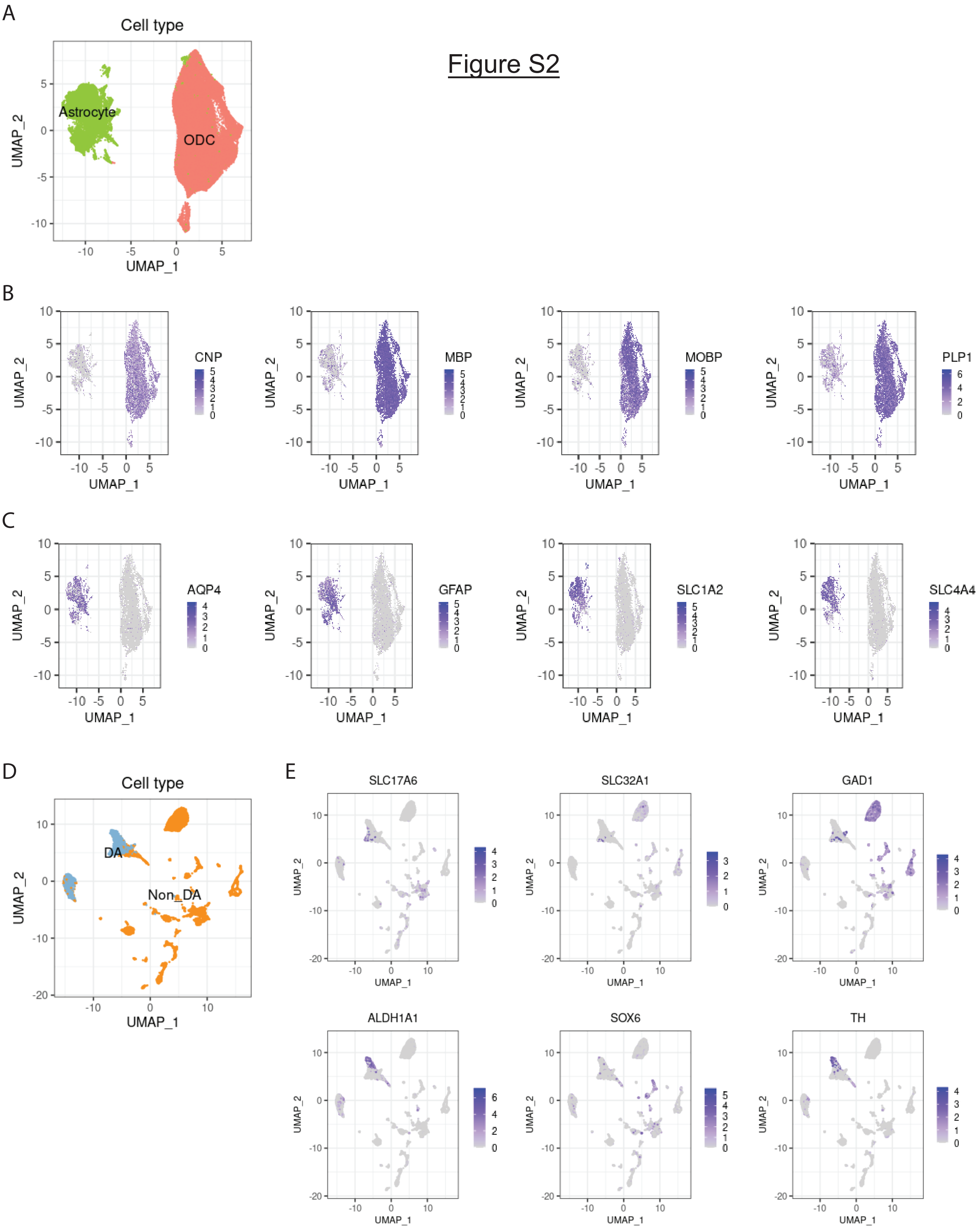
A
Cell type
Astrocyte
ODC
UMAP_1
UMAP_2
B
CNP
MBP
MOBP
PLP1
C
AQP4
GFAP
SLC1A2
SLC4A4
D
Cell type
DA
Non_DA
E
SLC17A6
SLC32A1
GAD1
ALDH1A1
SOX6
TH

Figure S2: Distribution of single nuclei with astrocyte, oligodendrocyte (ODC) and neuronal gene expression. UMAP plots showing (A) separation of astrocyte and ODC nuclei, (B,C) expression of key (B) ODC marker genes, essentially limited to nuclei in ODC cluster, (C) astrocyte marker genes essentially limited to nuclei in the astrocyte cluster, (D,E) separation of neuronal nuclei into dopaminergic and non-dopaminergic subtypes which can be further split into subpopulations of nuclei expressing gabaergic (*GAD1, SLC32A1*) and glutamatergic (*SLC17A6*) neuron marker genes (see text).

Figure S3

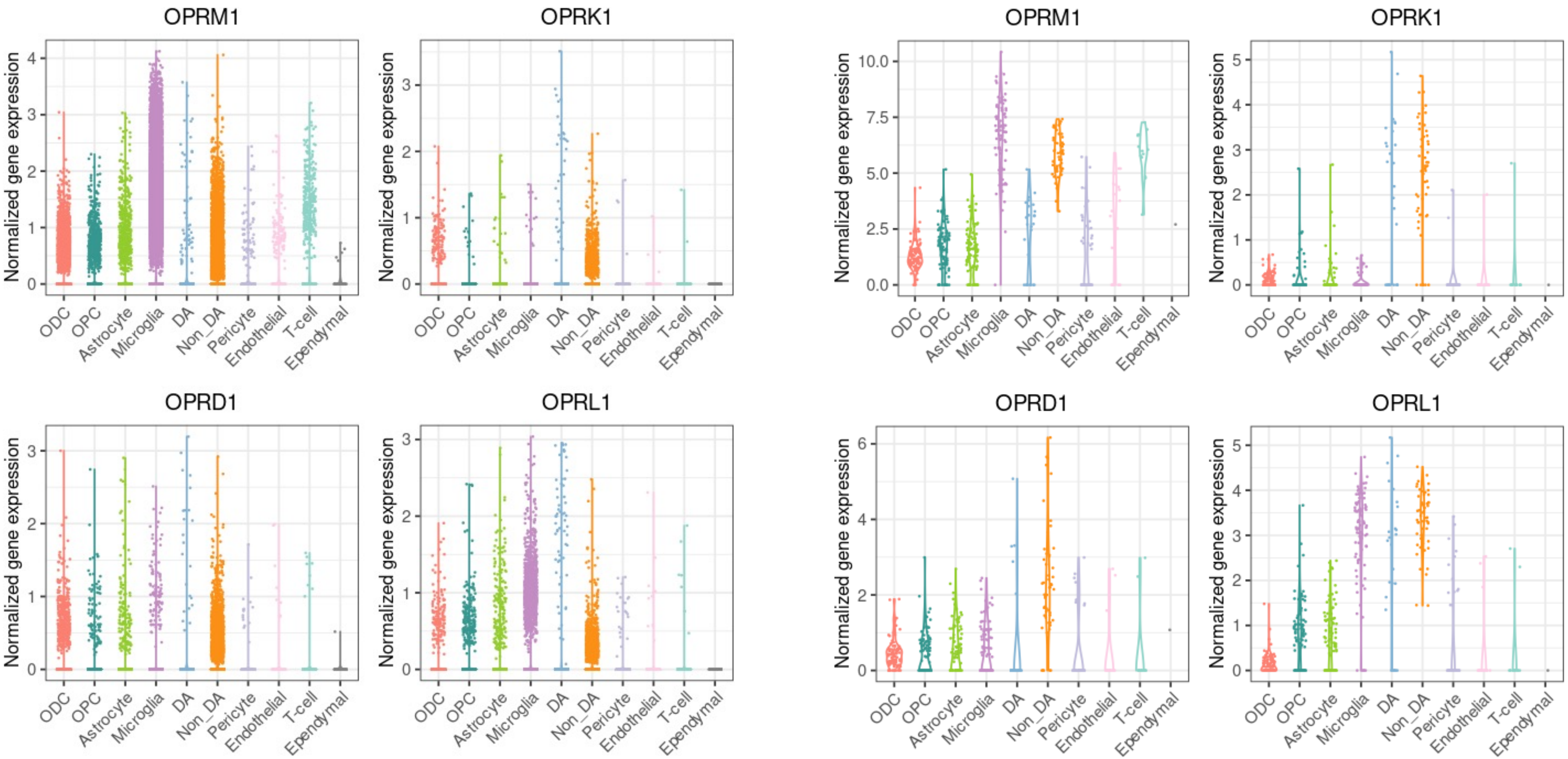
OPRM1
OPRK1
OPRD1
OPRL1
Normalized gene expression
ODC
OPC
Astrocyte
Microglia
DA
Non_DA
Pericyte
Endothelial
T-cell
Ependymal

Figure S3: Cell type-specific expression of opioid receptor genes in the VM. Violin plots depicting nuclear RNA levels for each of the 4 opioid receptor genes, *OPRM1, OPRK1, OPRD1 and OPRL*, for each of the 10 major VM cell types (x-axes) as indicated (left) at the single nucleus level (Seurat, default parameters) and (right) as cell type by subject, data shown as at normalized CPM, log2 transformed (+1).

Figure S4

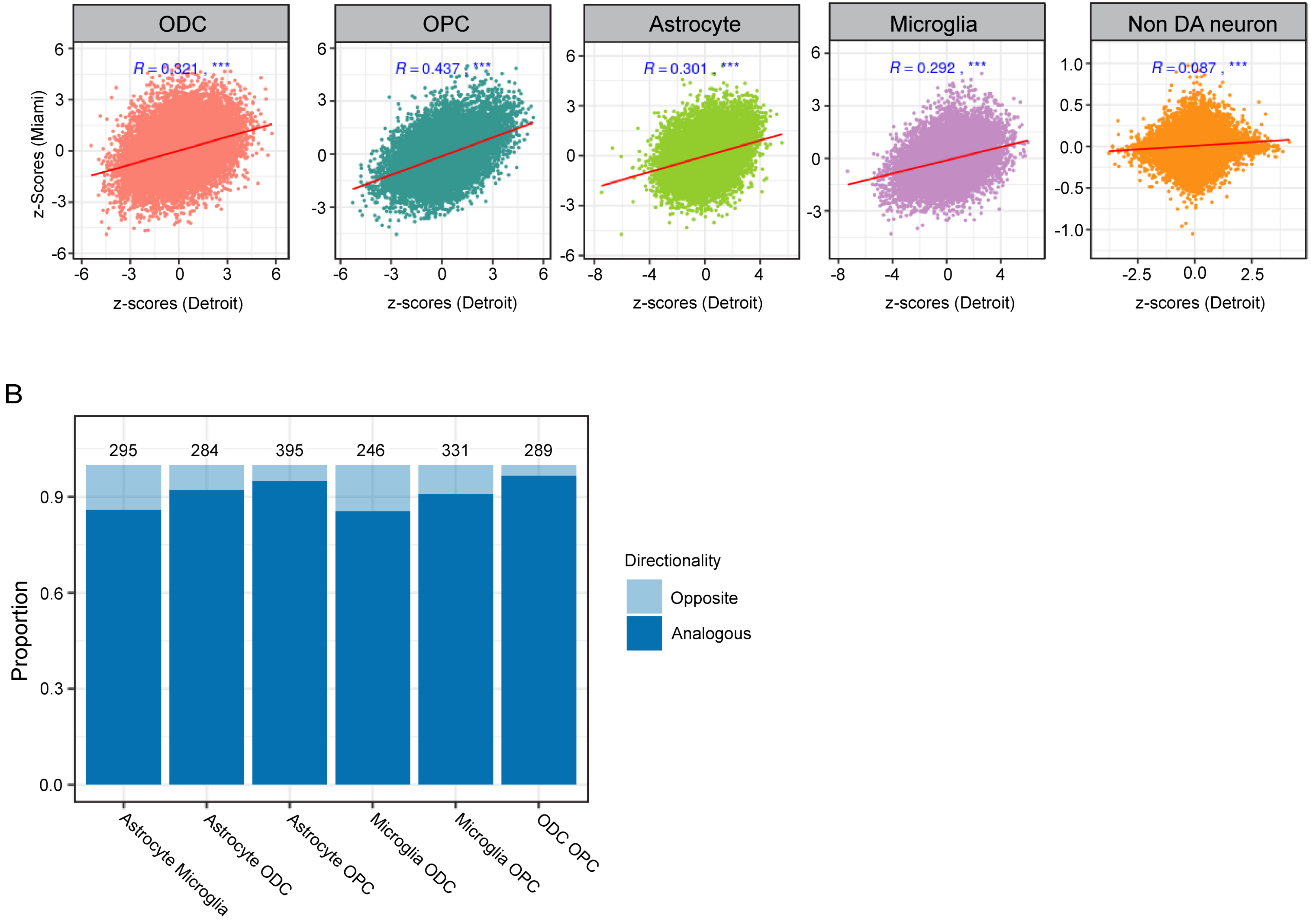
A
ODC
OPC
Astrocyte
Microglia
Non DA neuron
R = 0.321 , ***
R = 0.437 , ***
R = 0.301 , ***
R = 0.292 , ***
R = 0.087 , ***
z-Scores (Miami)
z-scores (Detroit)
B
Proportion
295
284
395
246
331
289
Directionality
Opposite
Analogous
Astrocyte Microglia
Astrocyte ODC
Astrocyte OPC
Microglia ODC
Microglia OPC
ODC OPC

Figure S4: **Differential gene expression across cohorts and cell types**. (A) Z-score (disease control differential) correlations comparing (y-axis, Miami cohort; x-axis Detroit cohort) cohorts for 4 major glial populations, as indicated, and non-dopaminergic neurons in the VM. (B) Proportional representation of DEGs shared between specific pairs of glial subtypes, as indicated on x-axis, based on shared vs. opposite directionality.

Figure S5

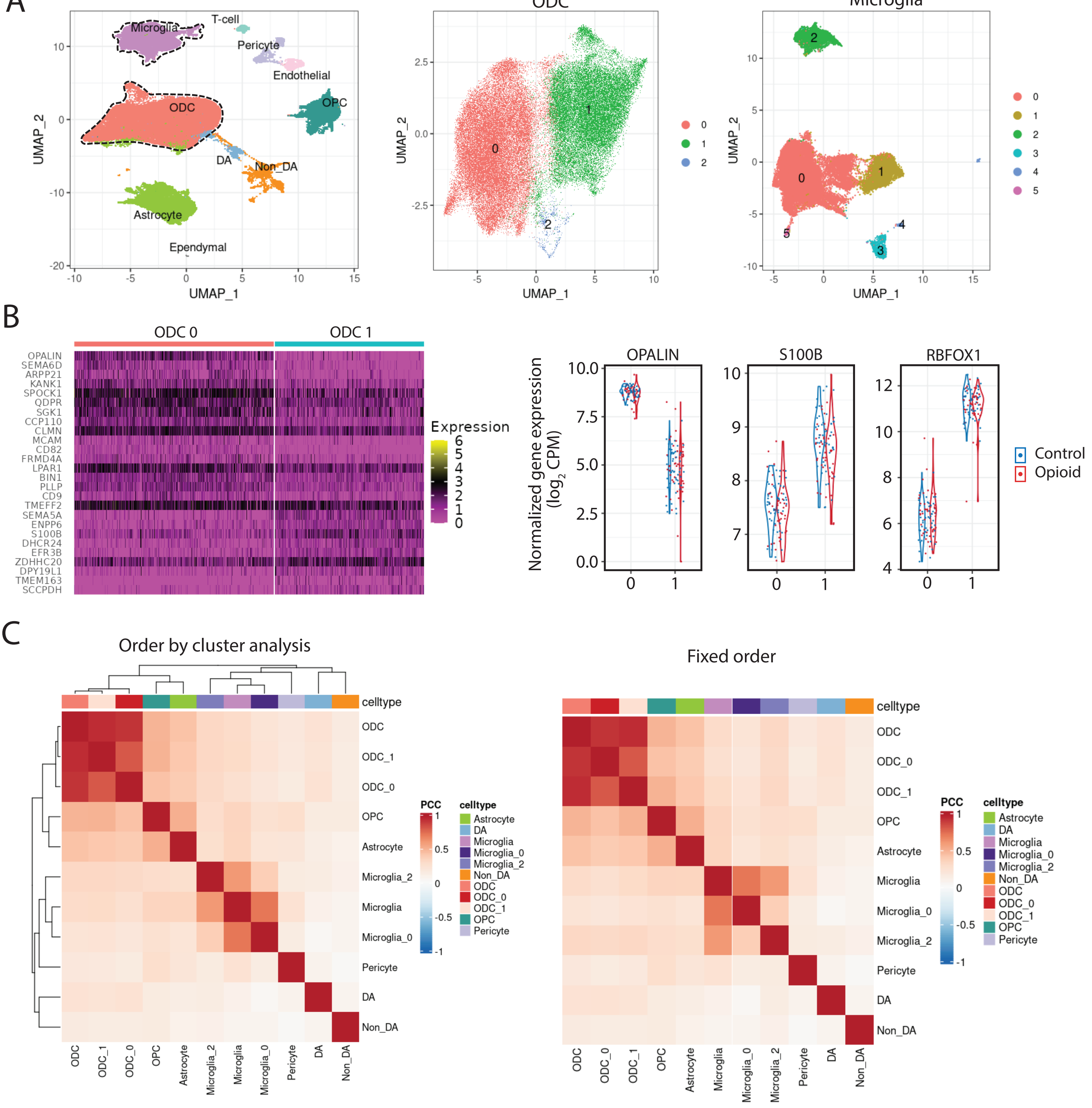

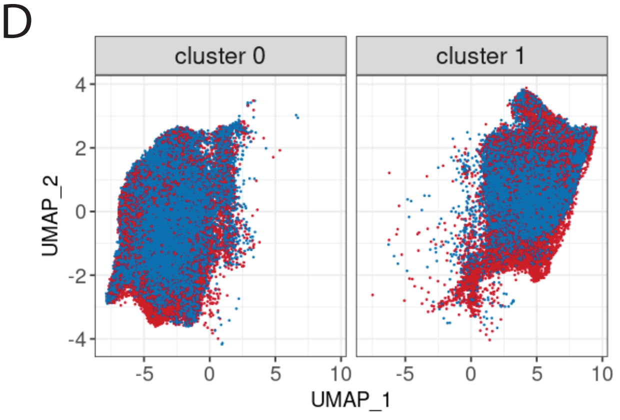

#Nuclei of Opioid and Control for each subcluster

	Opioid	Control
Cluster 0	26414	39177
Cluster 1	21696	27453

Figure S5: **Opioid exposure affects the transcriptome of multiple subtypes of oligodendrocytes and microglia.** (A, B) UMAP subclusterings for (A) oligodendrocyte and microglial populations. (B) (left) Differential genes for ODC subclusters ‘0’and ‘1, as indicated, (right) Violin plots summarizing *OPALIN, S100B* and *RBFOX1* marker gene expression levels by subject (midbrain sample) and oligodendrocyte subtype, as indicated (C) Correlational matrix for differential gene expression (diseased versus control individuals) across all glial and neuronal populations, including subtypes (D) population structure of ODC single nuclei, diseased individuals vs. controls, as indicated.

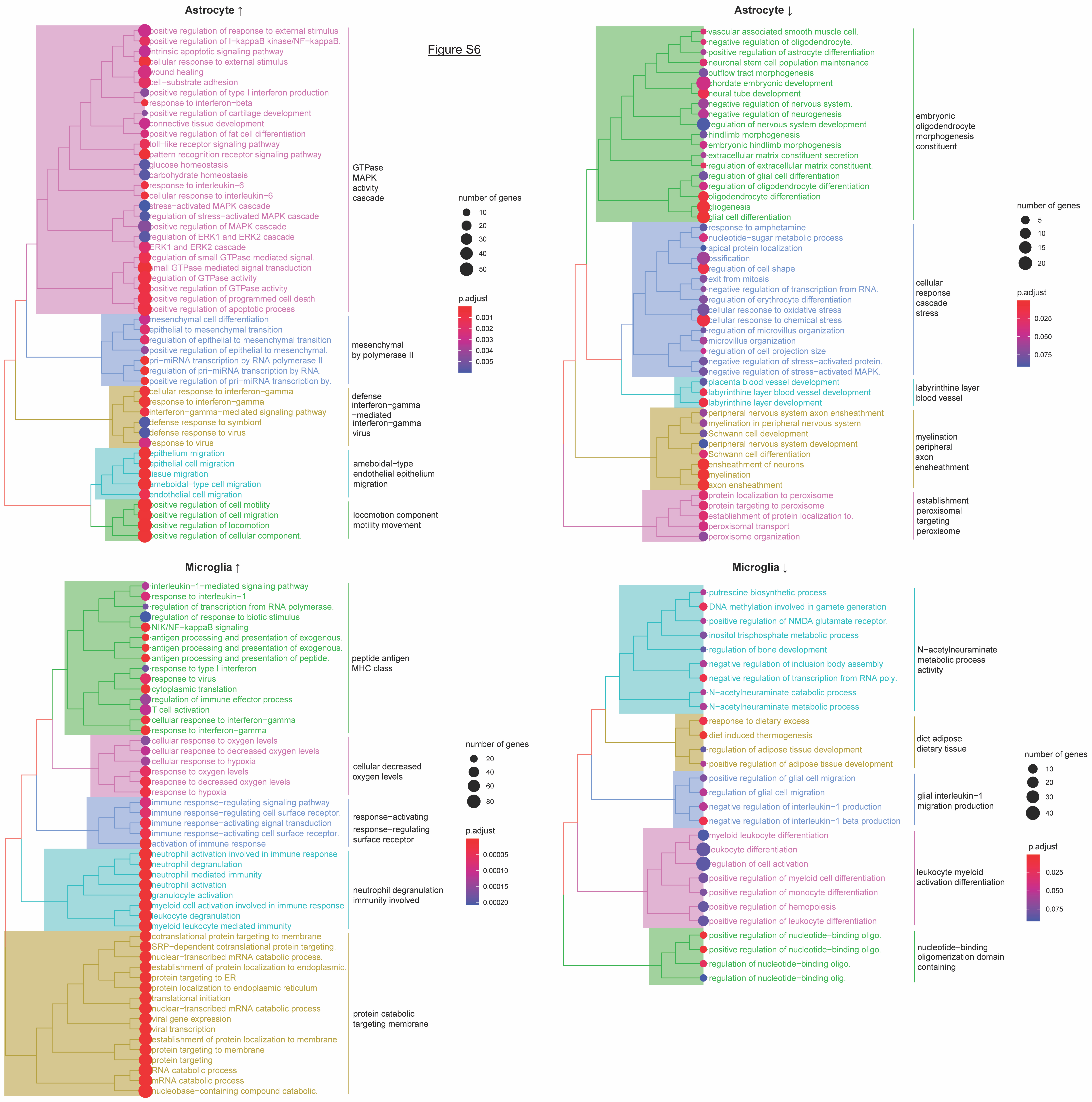
Figure S6
Astrocyte ↑
Astrocyte ↓
Microglia ↑
Microglia ↓
positive regulation of response to external stimulus
positive regulation of I−kappaB kinase/NF−kappaB.
intrinsic apoptotic signaling pathway
cellular response to external stimulus
wound healing
cell−substrate adhesion
positive regulation of type I interferon production
response to interferon−beta
positive regulation of cartilage development
connective tissue development
positive regulation of fat cell differentiation
toll−like receptor signaling pathway
pattern recognition receptor signaling pathway
glucose homeostasis
carbohydrate homeostasis
response to interleukin−6
cellular response to interleukin−6
stress−activated MAPK cascade
regulation of stress−activated MAPK cascade
positive regulation of MAPK cascade
regulation of ERK1 and ERK2 cascade
ERK1 and ERK2 cascade
regulation of small GTPase mediated signal.
small GTPase mediated signal transduction
regulation of GTPase activity
positive regulation of GTPase activity
positive regulation of programmed cell death
positive regulation of apoptotic process
mesenchymal cell differentiation
epithelial to mesenchymal transition
regulation of epithelial to mesenchymal transition
positive regulation of epithelial to mesenchymal.
pri−miRNA transcription by RNA polymerase II
regulation of pri−miRNA transcription by RNA.
positive regulation of pri−miRNA transcription by.
cellular response to interferon−gamma
response to interferon−gamma
interferon−gamma−mediated signaling pathway
defense response to symbiont
defense response to virus
response to virus
epithelium migration
epithelial cell migration
tissue migration
ameboidal−type cell migration
endothelial cell migration
positive regulation of cell motility
positive regulation of cell migration
positive regulation of locomotion
positive regulation of cellular component.
GTPase
MAPK
activity
cascade
mesenchymal
by polymerase II
defense
interferon−gamma
−mediated
interferon−gamma
virus
ameboidal−type
endothelial epithelium
migration
locomotion component
motility movement
number of genes
10
20
30
40
50
p.adjust
0.001
0.002
0.003
0.004
0.005
vascular associated smooth muscle cell.
negative regulation of oligodendrocyte.
positive regulation of astrocyte differentiation
neuronal stem cell population maintenance
outflow tract morphogenesis
chordate embryonic development
neural tube development
negative regulation of nervous system.
negative regulation of neurogenesis
regulation of nervous system development
hindlimb morphogenesis
embryonic hindlimb morphogenesis
extracellular matrix constituent secretion
regulation of extracellular matrix constituent.
regulation of glial cell differentiation
regulation of oligodendrocyte differentiation
oligodendrocyte differentiation
gliogenesis
glial cell differentiation
response to amphetamine
nucleotide−sugar metabolic process
apical protein localization
ossification
regulation of cell shape
exit from mitosis
negative regulation of transcription from RNA.
regulation of erythrocyte differentiation
cellular response to oxidative stress
cellular response to chemical stress
regulation of microvillus organization
microvillus organization
regulation of cell projection size
negative regulation of stress−activated protein.
negative regulation of stress−activated MAPK.
placenta blood vessel development
labyrinthine layer blood vessel development
labyrinthine layer development
peripheral nervous system axon ensheathment
myelination in peripheral nervous system
Schwann cell development
peripheral nervous system development
Schwann cell differentiation
ensheathment of neurons
myelination
axon ensheathment
protein localization to peroxisome
protein targeting to peroxisome
establishment of protein localization to.
peroxisomal transport
peroxisome organization
embryonic
oligodendrocyte
morphogenesis
constituent
cellular
response
cascade
stress
labyrinthine layer
blood vessel
myelination
peripheral
axon
ensheathment
establishment
peroxisomal
targeting
peroxisome
number of genes
5
10
15
20
p.adjust
0.025
0.050
0.075
interleukin−1−mediated signaling pathway
response to interleukin−1
regulation of transcription from RNA polymerase.
regulation of response to biotic stimulus
NIK/NF−kappaB signaling
antigen processing and presentation of exogenous.
antigen processing and presentation of exogenous.
antigen processing and presentation of peptide.
response to type I interferon
response to virus
cytoplasmic translation
regulation of immune effector process
T cell activation
cellular response to interferon−gamma
response to interferon−gamma
cellular response to oxygen levels
cellular response to decreased oxygen levels
cellular response to hypoxia
response to oxygen levels
response to decreased oxygen levels
response to hypoxia
immune response−regulating signaling pathway
immune response−regulating cell surface receptor.
immune response−activating signal transduction
immune response−activating cell surface receptor.
activation of immune response
neutrophil activation involved in immune response
neutrophil degranulation
neutrophil mediated immunity
neutrophil activation
granulocyte activation
myeloid cell activation involved in immune response
leukocyte degranulation
myeloid leukocyte mediated immunity
cotranslational protein targeting to membrane
SRP−dependent cotranslational protein targeting.
nuclear−transcribed mRNA catabolic process.
establishment of protein localization to endoplasmic.
protein targeting to ER
protein localization to endoplasmic reticulum
translational initiation
nuclear−transcribed mRNA catabolic process
viral gene expression
viral transcription
establishment of protein localization to membrane
protein targeting to membrane
protein targeting
RNA catabolic process
mRNA catabolic process
nucleobase−containing compound catabolic.
peptide antigen
MHC class
cellular decreased
oxygen levels
response−activating
response−regulating
surface receptor
neutrophil degranulation
immunity involved
protein catabolic
targeting membrane
number of genes
20
40
60
80
p.adjust
0.00005
0.00010
0.00015
0.00020
putrescine biosynthetic process
DNA methylation involved in gamete generation
positive regulation of NMDA glutamate receptor.
inositol trisphosphate metabolic process
regulation of bone development
negative regulation of inclusion body assembly
negative regulation of transcription from RNA poly.
N−acetylneuraminate catabolic process
N−acetylneuraminate metabolic process
response to dietary excess
diet induced thermogenesis
regulation of adipose tissue development
positive regulation of adipose tissue development
positive regulation of glial cell migration
regulation of glial cell migration
negative regulation of interleukin−1 production
negative regulation of interleukin−1 beta production
myeloid leukocyte differentiation
leukocyte differentiation
regulation of cell activation
positive regulation of myeloid cell differentiation
positive regulation of monocyte differentiation
positive regulation of hemopoiesis
positive regulation of leukocyte differentiation
positive regulation of nucleotide−binding oligo.
positive regulation of nucleotide−binding oligo.
regulation of nucleotide−binding oligo.
regulation of nucleotide−binding olig.
N−acetylneuraminate
metabolic process
activity
diet adipose
dietary tissue
glial interleukin−1
migration production
leukocyte myeloid
activation differentiation
nucleotide−binding
oligomerization domain
containing
number of genes
10
20
30
40
p.adjust
0.025
0.050
0.075

Figure S6: Cell-type specific pathway enrichments of differentially expressed genes in astrocytes and microglia. GO pathway enrichments for Biological Process, shown separately for up- and down-regulated DEGs, as indicated. See also Figure 3 and Data S5, including gene ratios (Data S5, column E).

Figure S7

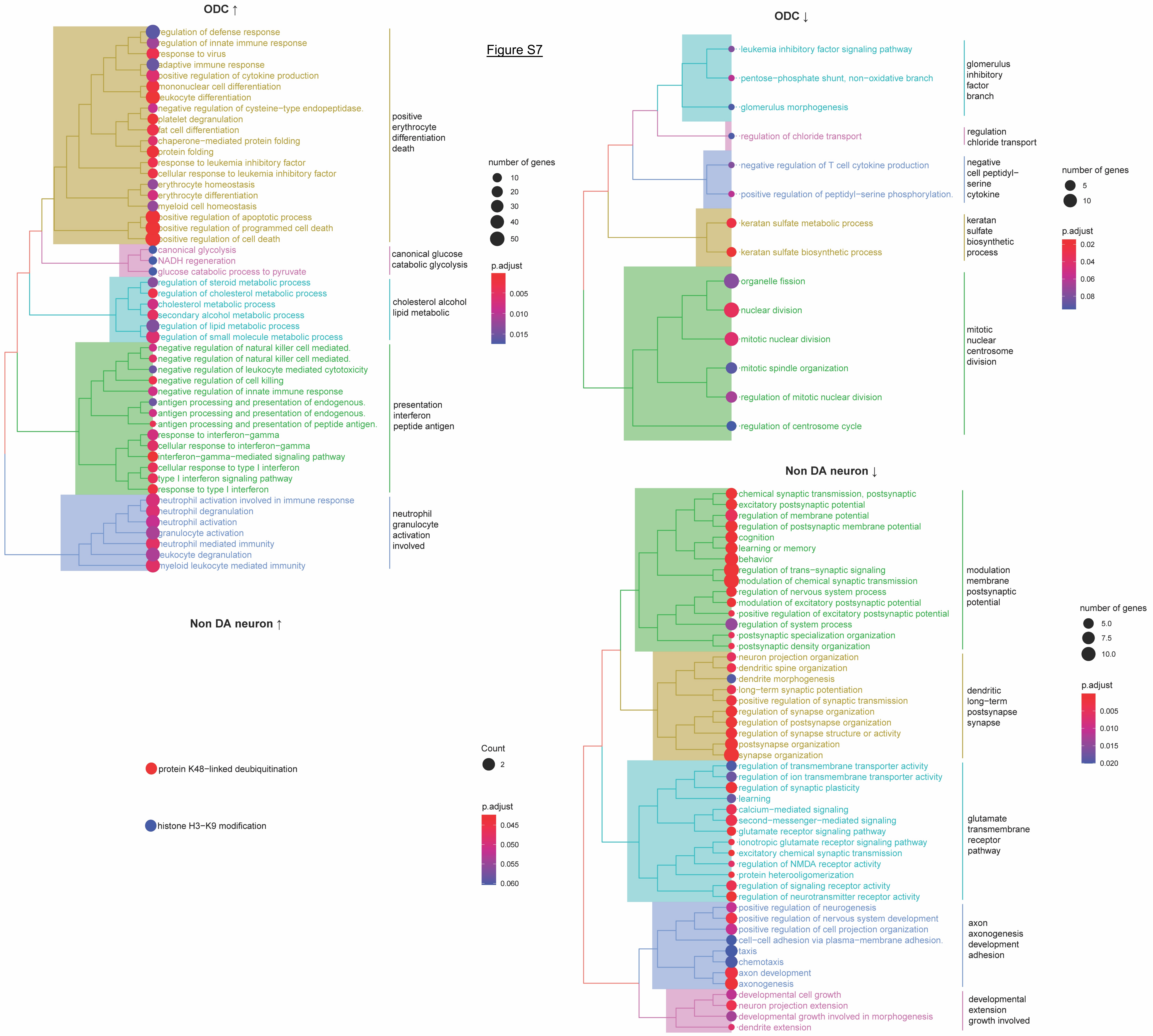

ODC ↑
regulation of defense response
regulation of innate immune response
response to virus
adaptive immune response
positive regulation of cytokine production
mononuclear cell differentiation
leukocyte differentiation
negative regulation of cysteine−type endopeptidase.
platelet degranulation
fat cell differentiation
chaperone−mediated protein folding
protein folding
response to leukemia inhibitory factor
cellular response to leukemia inhibitory factor
erythrocyte homeostasis
erythrocyte differentiation
myeloid cell homeostasis
positive regulation of apoptotic process
positive regulation of programmed cell death
positive regulation of cell death
canonical glycolysis
NADH regeneration
glucose catabolic process to pyruvate
regulation of steroid metabolic process
regulation of cholesterol metabolic process
cholesterol metabolic process
secondary alcohol metabolic process
regulation of lipid metabolic process
regulation of small molecule metabolic process
negative regulation of natural killer cell mediated.
negative regulation of natural killer cell mediated.
negative regulation of leukocyte mediated cytotoxicity
negative regulation of cell killing
negative regulation of innate immune response
antigen processing and presentation of endogenous.
antigen processing and presentation of endogenous.
antigen processing and presentation of peptide antigen.
response to interferon−gamma
cellular response to interferon−gamma
interferon−gamma−mediated signaling pathway
cellular response to type I interferon
type I interferon signaling pathway
response to type I interferon
neutrophil activation involved in immune response
neutrophil degranulation
neutrophil activation
granulocyte activation
neutrophil mediated immunity
leukocyte degranulation
myeloid leukocyte mediated immunity
positive erythrocyte differentiation death
canonical glucose catabolic glycolysis
cholesterol alcohol lipid metabolic
presentation interferon peptide antigen
neutrophil granulocyte activation involved
number of genes
10
20
30
40
50
p.adjust
0.005
0.010
0.015
ODC ↓
leukemia inhibitory factor signaling pathway
pentose−phosphate shunt, non−oxidative branch
glomerulus morphogenesis
regulation of chloride transport
negative regulation of T cell cytokine production
positive regulation of peptidyl−serine phosphorylation.
keratan sulfate metabolic process
keratan sulfate biosynthetic process
organelle fission
nuclear division
mitotic nuclear division
mitotic spindle organization
regulation of mitotic nuclear division
regulation of centrosome cycle
glomerulus inhibitory factor branch
regulation chloride transport
negative cell peptidyl− serine cytokine
keratan sulfate biosynthetic process
mitotic nuclear centrosome division
number of genes
5
10
p.adjust
0.02
0.04
0.06
0.08
Non DA neuron ↑
protein K48−linked deubiquitination
histone H3−K9 modification
Count
2
p.adjust
0.045
0.050
0.055
0.060
Non DA neuron ↓
chemical synaptic transmission, postsynaptic
excitatory postsynaptic potential
regulation of membrane potential
regulation of postsynaptic membrane potential
cognition
learning or memory
behavior
regulation of trans−synaptic signaling
modulation of chemical synaptic transmission
regulation of nervous system process
modulation of excitatory postsynaptic potential
positive regulation of excitatory postsynaptic potential
regulation of system process
postsynaptic specialization organization
postsynaptic density organization
neuron projection organization
dendritic spine organization
dendrite morphogenesis
long−term synaptic potentiation
positive regulation of synaptic transmission
regulation of synapse organization
regulation of postsynapse organization
regulation of synapse structure or activity
postsynapse organization
synapse organization
regulation of transmembrane transporter activity
regulation of ion transmembrane transporter activity
regulation of synaptic plasticity
learning
calcium−mediated signaling
second−messenger−mediated signaling
glutamate receptor signaling pathway
ionotropic glutamate receptor signaling pathway
excitatory chemical synaptic transmission
regulation of NMDA receptor activity
protein heterooligomerization
regulation of signaling receptor activity
regulation of neurotransmitter receptor activity
positive regulation of neurogenesis
positive regulation of nervous system development
positive regulation of cell projection organization
cell−cell adhesion via plasma−membrane adhesion.
taxis
chemotaxis
axon development
axonogenesis
developmental cell growth
neuron projection extension
developmental growth involved in morphogenesis
dendrite extension
modulation membrane postsynaptic potential
dendritic long−term postsynapse synapse
glutamate transmembrane receptor pathway
axon axonogenesis development adhesion
developmental extension growth involved
number of genes
5.0
7.5
10.0
p.adjust
0.005
0.010
0.015
0.020

Figure S7: Cell-type specific pathway enrichments of differentially expressed genes in oligodendrocytes (ODC) **and non-dopaminergic neurons** (Non-DA)**.** GO pathway enrichments for Biological Process, shown separately for up- and down-regulated, as indicated. See also Figure 3 and Data S5, including gene ratios (Data S5, column E).

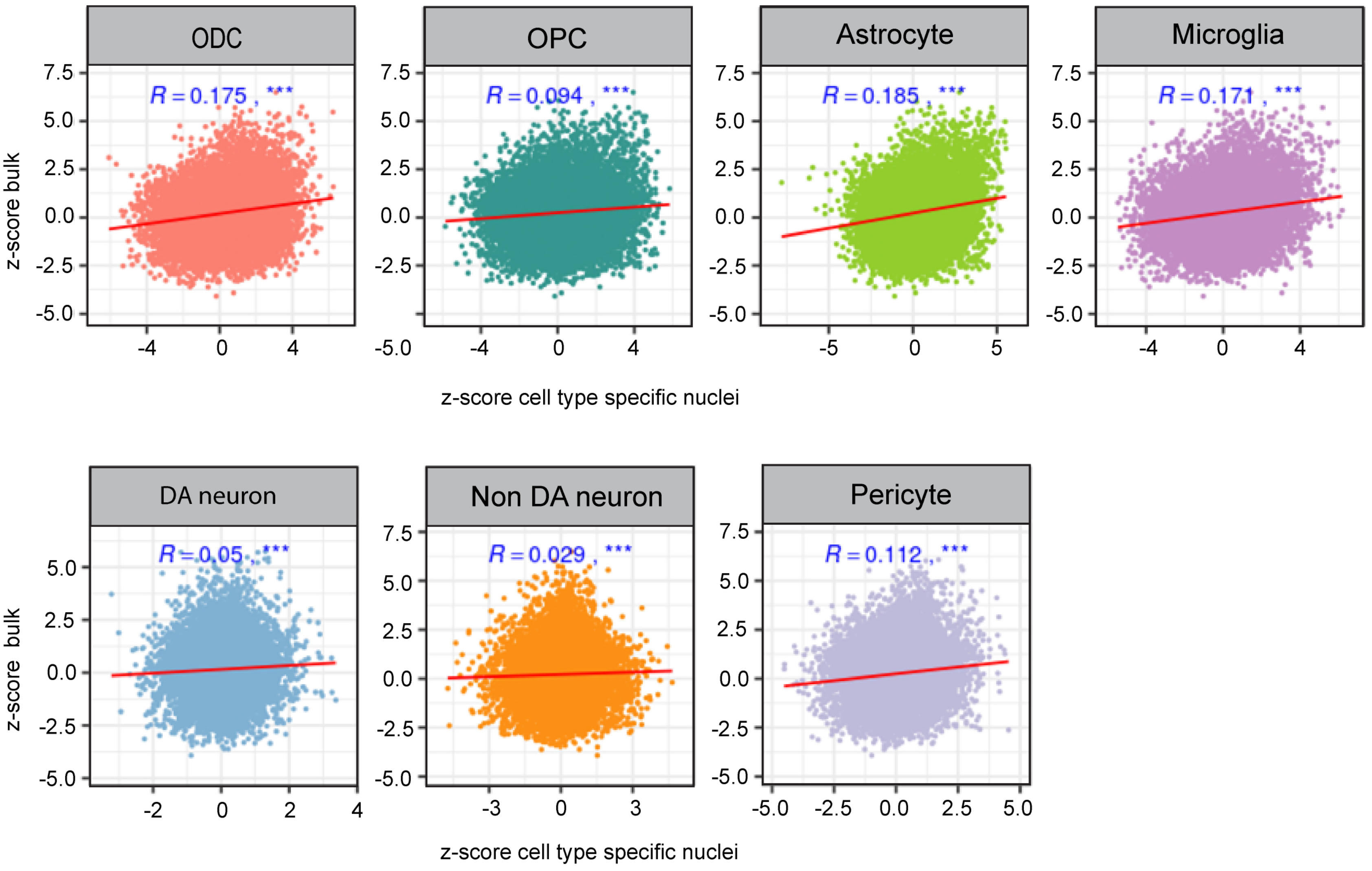
ODC
R = 0.175 , ***
OPC
R = 0.094 , ***
Astrocyte
R = 0.185 , ***
Microglia
R = 0.171 , ***
DA neuron
R = 0.05 , ***
Non DA neuron
R = 0.029 , ***
Pericyte
R = 0.112 , ***
z-score bulk
z-score cell type specific nuclei

Figure S8

Figure S8: **Correlations between prior bulk RNA-seq study and the current RNA-seq analysis with single cell resolution.** Z-score (disease vs. control differential) correlations comparing (y-axis, bulk tissue RNA-seq study; x-axis, cell type specific RNA-seq at single nuclei resolution, current study) for 7 cell types, as indicated.

Figure S9

A

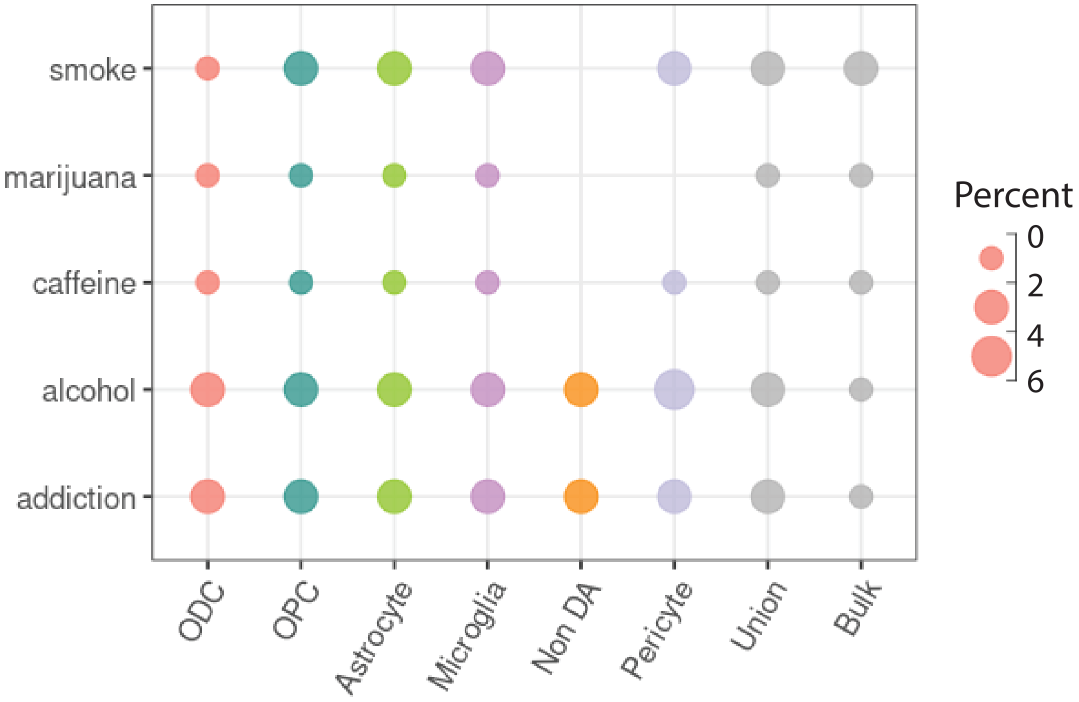

smoke
marijuana
caffeine
alcohol
addiction
ODC
OPC
Astrocyte
Microglia
Non DA
Pericyte
Union
Bulk
Percent
0
2
4
6

B

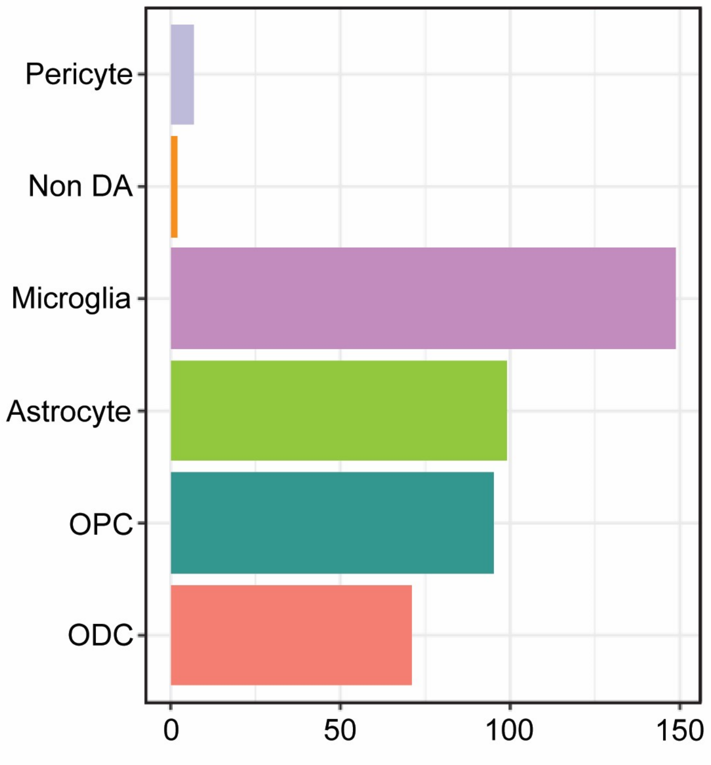

Pericyte
Non DA
Microglia
Astrocyte
OPC
ODC
0
50
100
150

Figure S9: **DEGs linked to TWAS**. (A) Percentages representing, for each cell type the normalized proportion of DEGs overlapping with PhenomeXcan TWAS for substance use and medical or neurological traits, as indicated. We focused on Substantia Nigra (SN) gene expression and population-scale SUD phenotypes in PhenomeXcan. We included a total of 1,260 PhenomeXcan genes for a total of 40 SUD-related traits from the following categories of traits or diseases: alcohol, caffeine, marijuana, and smoking (*Data S6*). We also analyzed a recent study[2] that had called addiction risk genes via TWAS-guided integration of GTEX and PsychENCODE expression quantitative trait loci (see Data S7,S8). For GTEx, the study conducted TWAS analyses using MetaXcan via integration of eQTL from 13 brain regions and identified a total of 351 addiction risk factor genes (FDR<10%). For PsychENCODE, using the frontal and temporal cortex, TWAS analysis using S-PrediXcan identified a total of 410 addiction risk genes with FDR<10%[2]. (B) Cell-type specific counts of genes called as DEG in current study *and* linked to substance use trait(s) in PhenomeXcan (see Data S7).

Figure S10

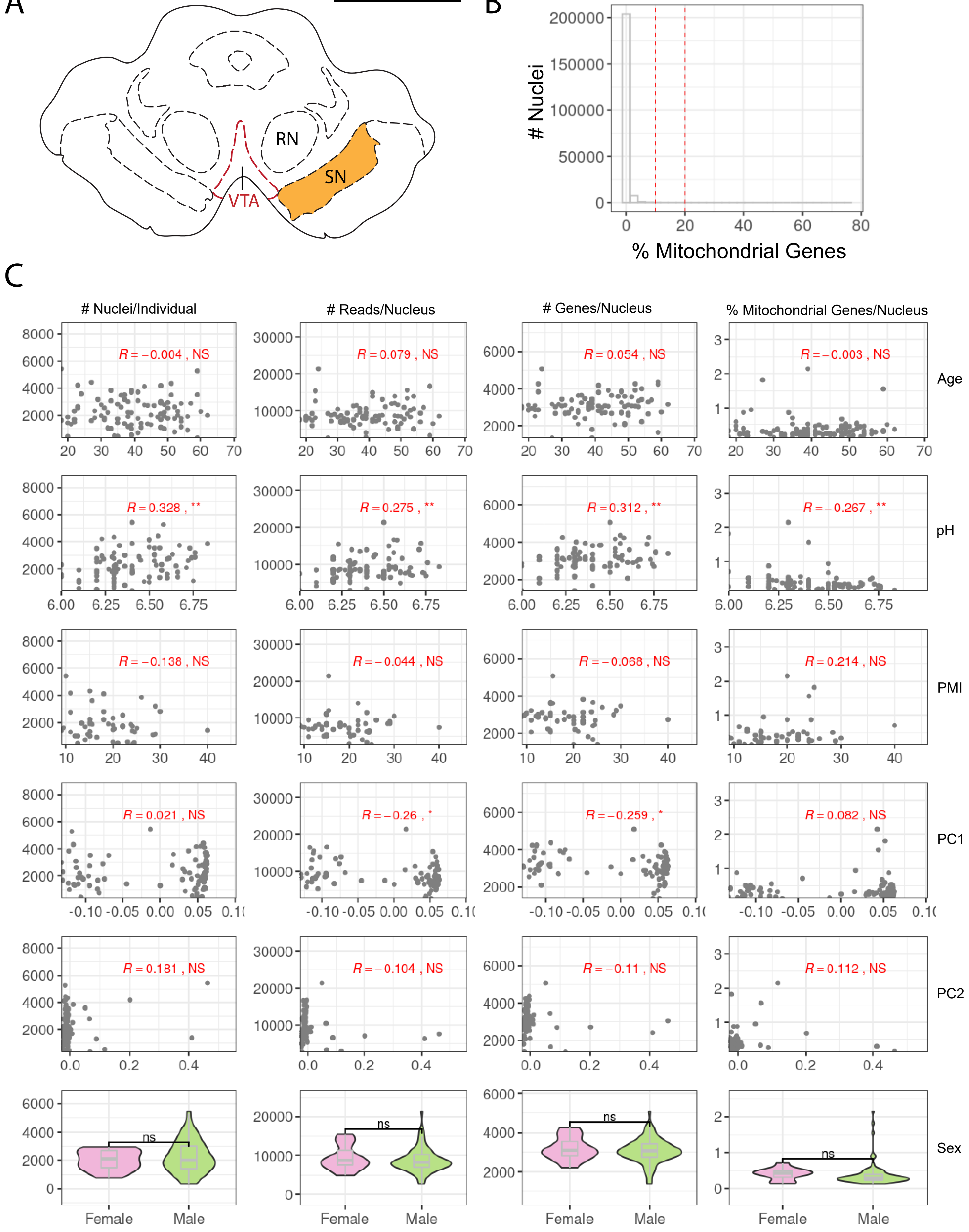

A
RN
SN
VTA
B
Nuclei
% Mitochondrial Genes
C
Nuclei/Individual
Reads/Nucleus
Genes/Nucleus
% Mitochondrial Genes/Nucleus
R = −0.004 , NS
R = 0.079 , NS
R = 0.054 , NS
R = −0.003 , NS
Age
R = 0.328 , **
R = 0.275 , **
R = 0.312 , **
R = −0.267 , **
pH
R = −0.138 , NS
R = −0.044 , NS
R = −0.068 , NS
R = 0.214 , NS
PMI
R = 0.021 , NS
R = −0.26 , *
R = −0.259 , *
R = 0.082 , NS
PC1
R = 0.181 , NS
R = −0.104 , NS
R = −0.11 , NS
R = 0.112 , NS
PC2
ns
Female
Male
Sex

Figure S10: Tissue and demographic variables impacting single nuclei transcriptomes (A) Ventral midbrain region-of-interest. Drawing from representative coronally cut VM tissue block, showing the substantia nigra and adjacent portion of ventral tegmental area, the region-of-interest prepared by dissection and then further processed with the nuclei extraction protocol (see Methods). (B, C) Effects of tissue quality and demographic variables on single nuclei transcriptomes. (B) Frequency histogram, showing the fraction of mitochondrial genes/nucleus. Note fraction of <1% for the overwhelming majority, or 93.7% of nuclei. Note also that there are 1.8% of nuclei with >2% mitochondrial, 0.1% of nuclei with >10% mitochondrial and 0.025% of nuclei had >20% mitochondrial reads. (C) Linear regression testing for association of (left to right) nuclei number/donor, number of reads/nuclei, number of genes/nucleus and fraction of mitochondrial reads/nucleus with (top to bottom) donor age, brain tissue pH, postmortem interval (PMI), genotype principal component PC1 and PC2, and sex, *(**) P< 0.05, 0.01.

Table S1: Summary of Case and Control Cohort (Demographics and postmortem confounds)

Cohort	Category	N	Age (yrs)	Sex (M:F)	Race (W:AA:H)	PMI (hrs)	pH
Detroit	Opioid	22	43.86 ± 11.66	18:4	13:9:0	<20	6.46 ± 0.16
	Control	22	43.55 ± 9.39	18:4	13:9:0	<20	6.54 ± 0.20
Miami	Opioid	23	36.30 ± 9.04	20:3	18:3:2	20.91 ± 5.56	6.27 ± 0.14
	Control	28	37.21 ± 12.82	28:0	17:9:2	16.44 ± 5.04	6.34 ± 0.16

Supplemental References

1. Kamath T, *et al.* Single-cell genomic profiling of human dopamine neurons identifies a population that selectively degenerates in Parkinson's disease. *Nat Neurosci* **25**, 588-595 (2022).

2. Hatoum AS, *et al.* Multivariate genome-wide association meta-analysis of over 1 million subjects identifies loci underlying multiple substance use disorders. *Nat Ment Health* **1**, 210-223 (2023).
