# Supplementary material for: Single nucleus transcriptomics of ventral midbrain identifies glial activation associated with chronic opioid use disorder
Source: Nat Commun. 2023 Sep 12;14:5610. doi: 10.1038/s41467-023-41455-8 (PMC10497570; doi:10.1038/s41467-023-41455-8)
Supplement: Supplementary file 3 — Description of Additional Supplementary Files [file 41467_2023_41455_MOESM3_ESM.pdf]

Description of Additional Supplementary Files

File Name: Supplementary Data 1
Description: Brain donor metadata.

File Name: Supplementary Data 2
Description: Sample by sample numbers of reads, genes, and nuclei per cell type.

File Name: Supplementary Data 3
Description: Differentially expressed genes by cell type.

File Name: Supplementary Data 4
Description: Top 100 differentially expressed genes in subclusters of microglia and oligodendrocytes.

File Name: Supplementary Data 5
Description: Gene Ontologies (Biological Process) of Differentially Expressed Genes by Cell Type.

File Name: Supplementary Data 6
Description: Substance Use Traits in PhenomeXcan.

File Name: Supplementary Data 7
Description: Differentially expressed genes that match to Substance Use Associated Variants in PhenomeXcan.

File Name: Supplementary Data 8
Description: Differential expression and substance use traits (TWAS), PhenomeXScan and Hatoum et al. (GTEX, Psychencode) 2023.

File Name: Supplementary Data 9
Description: Differentially expressed genes in present study matching to differentially expressed genes reported in mouse model for opiate exposure.

File Name: Supplementary Data 10
Description: Differentially expressed neuronal gene in present study matching to locus with altered histone acetylation in prefrontal cortex of individuals who died by opioid overdose.
