# Supplementary material for: Single nucleus transcriptomics of ventral midbrain identifies glial activation associated with chronic opioid use disorder
Source: Nat Commun. 2023 Sep 12;14:5610. doi: 10.1038/s41467-023-41455-8 (PMC10497570; doi:10.1038/s41467-023-41455-8)
Supplement: Supplementary file 14 — Reporting Summary [file 41467_2023_41455_MOESM14_ESM.pdf]

nature portfolio

Corresponding author(s): Schahram Akbarian

Last updated by author(s): 08-14-23

Reporting Summary

Nature Portfolio wishes to improve the reproducibility of the work that we publish. This form provides structure for consistency and transparency in reporting. For further information on Nature Portfolio policies, see our Editorial Policies and the Editorial Policy Checklist.

Statistics

For all statistical analyses, confirm that the following items are present in the figure legend, table legend, main text, or Methods section.

n/a	Confirmed	
☐	☒	The exact sample size (*n*) for each experimental group/condition, given as a discrete number and unit of measurement
☐	☒	A statement on whether measurements were taken from distinct samples or whether the same sample was measured repeatedly
☐	☒	The statistical test(s) used AND whether they are one- or two-sided<br>*Only common tests should be described solely by name; describe more complex techniques in the Methods section.*
☐	☒	A description of all covariates tested
☐	☒	A description of any assumptions or corrections, such as tests of normality and adjustment for multiple comparisons
☐	☒	A full description of the statistical parameters including central tendency (e.g. means) or other basic estimates (e.g. regression coefficient) AND variation (e.g. standard deviation) or associated estimates of uncertainty (e.g. confidence intervals)
☐	☒	For null hypothesis testing, the test statistic (e.g. *F*, *t*, *r*) with confidence intervals, effect sizes, degrees of freedom and *P* value noted<br>*Give P values as exact values whenever suitable.*
☒	☐	For Bayesian analysis, information on the choice of priors and Markov chain Monte Carlo settings
☒	☐	For hierarchical and complex designs, identification of the appropriate level for tests and full reporting of outcomes
☒	☐	Estimates of effect sizes (e.g. Cohen's *d*, Pearson's *r*), indicating how they were calculated

Our web collection on statistics for biologists contains articles on many of the points above.

Software and code

Policy information about availability of computer code

Data collection	10x genomics single cell nucleus RNA-sequencing
Data analysis	cellranger (V7.0) count using the GRCh38 human reference demuxlet<br>Seurat R package (v4.0) RunHarmony DESeq2/1.28.1<br>ClusterProfiler (4.0) R PhenomeXcan<br>Original code and scripts used to analyze the data is available on GitHub https://github.com/piquelab/sc_brains

For manuscripts utilizing custom algorithms or software that are central to the research but not yet described in published literature, software must be made available to editors and reviewers. We strongly encourage code deposition in a community repository (e.g. GitHub). See the Nature Portfolio guidelines for submitting code & software for further information.

Data

Policy information about availability of data

All manuscripts must include a data availability statement. This statement should provide the following information, where applicable:

- Accession codes, unique identifiers, or web links for publicly available datasets
- A description of any restrictions on data availability
- For clinical datasets or third party data, please ensure that the statement adheres to our policy

Annotated single nuclei RNA-seq data generated in this study have been deposited in the Gene Expression Omnibus (GEO) database under accession code GSE240457 [https://www.ncbi.nlm.nih.gov/geo/query/acc.cgi?acc=GSE240457]. The raw data are, per NIH Genomic Data Sharing Policy, available under restricted access in the database for Genotypes and Phenotypes (dbGAP) under accession code phs003260.v1.p1.

Research involving human participants, their data, or biological material

Policy information about studies with human participants or human data. See also policy information about sex, gender (identity/presentation), and sexual orientation and race, ethnicity and racism.

Reporting on sex and gender	All specimens were collected postmortem. No self-reports. Biological sex was confirmed by transcriptomic signal from the sex chromosomes.
Reporting on race, ethnicity, or other socially relevant groupings	All specimens were collected postmortem. No self-reports.
Population characteristics	Case and control brains were collected within two separate geographical areas of the U.S., representing the greater Detroit and Miami metropolitan areas. The two collection sites operated independently. Cause of death was determined by forensic pathologists following medico-legal investigations evaluating the circumstances of death including medical records, police reports and scene investigations, autopsy results, and toxicological data.
Recruitment	Human midbrain specimens were collected during routine autopsy by the Wayne County Medical Examiner as part of the autopsy process mandated by the laws of the State of Michigan. All cases and controls in the Detroit cohort were de-identified specimens. Cases in Miami were selected from an opportunistic sample of opioid intoxication deaths defined by circumstances of death and forensic and supplemental toxicology data.
Ethics oversight	Detroit cohort, all studies were approved by the Institutional Review Board of Wayne State University, Miami cohort , all studies were approved by the Institutional Review Board of Nova Southeastern University, with next of kin consent.

Note that full information on the approval of the study protocol must also be provided in the manuscript.

Field-specific reporting

Please select the one below that is the best fit for your research. If you are not sure, read the appropriate sections before making your selection.

☒ Life sciences ☐ Behavioural & social sciences ☐ Ecological, evolutionary & environmental sciences

For a reference copy of the document with all sections, see nature.com/documents/nr-reporting-summary-flat.pdf

Life sciences study design

All studies must disclose on these points even when the disclosure is negative.

Sample size	The number of donors and replicates needed was calculated by considering pilot study effect size calculations, balancing minimization of technical variation and experimental costs.
Data exclusions	No single nuclei RNA-seq data were excluded
Replication	We studied two independent brain collections (Detroit and Miami, see text) Experimental findings for next-generation sequencing data obtained from the Detroit cohort were replicated with the Miami cohort, by assessing the correlation of gene expression changes for cases versus controls in each cohort. As a case control cohort design, the study therefore has a 1x replication built in.
Randomization	All donor samples were allocated into control and experimental groups and analyzed in parallel and blinded to control for covariates.
Blinding	All next-generation sequencing data, although unblinded, was processed using unbiased algorithms and scripts via the Unix , Phyton and R platforms.

Reporting for specific materials, systems and methods

We require information from authors about some types of materials, experimental systems and methods used in many studies. Here, indicate whether each material, system or method listed is relevant to your study. If you are not sure if a list item applies to your research, read the appropriate section before selecting a response.

Materials & experimental systems

n/a	Involved in the study
☒	☐ Antibodies
☒	☐ Eukaryotic cell lines
☒	☐ Palaeontology and archaeology
☒	☐ Animals and other organisms
☒	☐ Clinical data
☒	☐ Dual use research of concern
☒	☐ Plants

Methods

n/a	Involved in the study
☒	☐ ChIP-seq
☒	☐ Flow cytometry
☒	☐ MRI-based neuroimaging
